# Mechanistic insights into the impact of Cold Atmospheric Pressure Plasma on human epithelial cell lines

**DOI:** 10.1038/srep41163

**Published:** 2017-01-25

**Authors:** Marlène Dezest, Laurent Chavatte, Marion Bourdens, Damien Quinton, Mylène Camus, Luc Garrigues, Pascal Descargues, Stéphane Arbault, Odile Burlet-Schiltz, Louis Casteilla, Franck Clément, Valérie Planat, Anne-Laure Bulteau

**Affiliations:** 1 IPREM, UMR 5254, Université de Pau et des Pays de l’Adour, 64000, Pau, France; 2 STROMALAB, Université de Toulouse, CNRS ERL5311, EFS, INP-ENVT, UPS, INSERM U1031, BP31432 Toulouse cedex 4, France; 3 Univ. BORDEAUX, ISM. CNRS UMR 5255 NSysA group, ENSCBP, Pessac, 33607, France; 4 Institut de Pharmacologie et de Biologie Structurale, Université de Toulouse, CNRS, UPS, France; 5 Genoskin, Centre Pierre Potier-Oncopole, F-31106 Toulouse, France

## Abstract

Compelling evidence suggests that Cold Atmospheric Pressure Plasma (CAPP) has potential as a new cancer therapy. However, knowledge about cellular signaling events and toxicity subsequent to plasma treatment is still poorly documented. The aim of this study was to focus on the interaction between 3 different types of plasma (He, He-O_2_, He-N_2_) and human epithelial cell lines to gain better insight into plasma-cell interaction. We provide evidence that reactive oxygen and nitrogen species (RONS) are inducing cell death by apoptosis and that the proteasome, a major intracellular proteolytic system which is important for tumor cell growth and survival, is a target of (He or He-N_2_) CAPP. However, RONS are not the only actors involved in cell death; electric field and charged particles could play a significant role especially for He-O_2_ CAPP. By differential label-free quantitative proteomic analysis we found that CAPP triggers antioxidant and cellular defense but is also affecting extracellular matrix in keratinocytes. Moreover, we found that malignant cells are more resistant to CAPP treatment than normal cells. Taken together, our findings provide insight into potential mechanisms of CAPP-induced proteasome inactivation and the cellular consequences of these events.

One of the most promising applications of the cold atmospheric pressure plasmas (CAPPs) in medicine is associated with cancer therapies especially skin cancer such as melanoma and carcinomas with the highest therapy resistance^1^. CAPPs are partially ionized gases that are out of thermodynamic equilibrium. These excited gases contains free charges (electrons, ions), free radicals, excited molecules and photons (UV), and generate a transient electric field^2^,^3^. Their proportions vary between plasmas and depend on the gas used, the reactor design and the electrical set up. CAPPs generate various kinds of reactive oxygen and nitrogen species (RONS) including hydroxyl radical (OH), hydrogen peroxide (H_2_O_2_), ozone (O_3_), atomic oxygen (O), superoxide anion (O_2_^−^), nitric oxide (NO) and peroxynitrite anion (ONOO^−^), these are considered to be the most biologically relevant components of plasma. The RONS composition in CAPP can be altered by regulating the voltage, frequency, working and feeding gases, and humidity^4^. There are numerous studies showing how CAPPs influence cells at molecular, genetic and epigenetic levels^5^,^6^. Understanding the mechanism of CAPP-cell interactions is necessary and crucial to assure safety during CAPP treatment. Recent studies have shown that CAPPs preferentially activate various cell death modalities in cancer cell lines compared to their normal counterparts^7^,^8^,^9^,^10^. For example it has the ability to induce cell death in glioblastoma but has a less toxic effect on normal astrocytes^11^. The magnitude of apoptosis is strongly dependent of the investigated cell type^9^,^12^. The aim of a successful plasma treatment is killing the desired cells without harming the surrounding healthy tissue. The side-effects of CAPP on cell culture studies of normal keratinocytes have not yet been fully investigated, although keratinocytes of the epidermal layer are the cells most directly affected during CAPP treatment of the skin. Therefore molecular and cellular mechanisms of plasma–induced toxicity on the *in vitro* effects on keratinocytes have to be examined. A recent study on prostate cancer cells showed that the responses to CAPP treatment were common to both normal and cancer primary samples^13^. Because targeting cellular metabolism and protein homeostasis is currently another approach for selectively killing cancer cells, we wanted to know if the ubiquitin-proteasome system, an important regulator of cell growth and apoptosis, was a target of plasma treatment. The current study was undertaken to characterize the effects of CAPP on proteasome activity and to assess how alterations in proteasome function may contribute to cell death. More than 80% of cellular proteins are degraded through this pathway including those involved in a broad array of processes such as cell cycle, apoptosis, transcription, DNA repair, protein quality control and antigen presentation^14^,^15^. As cancer cells are more sensitive to proteasome inhibition than normal cells due to their elevated proliferation rates and the loss of translation quality control, the pharmacological targeting of proteasomal activities provides a new and promising avenue for basic and clinical research^15^. CAPPs are producing RONS that lead to the production of oxidized proteins which are preferentially degraded by the proteasome^16^. Thus, alteration in proteasome activity upon CAPP exposure would be expected to significantly impact a number of cellular events, thereby influencing the outcome of cold plasma treatment.

We choose epithelial cell lines (human keratinocytes, human fibroblasts, human colorectal carcinoma and skin melanoma) to gain insight into plasma-cell interaction and to determine which cellular pathways are induced by CAPP treatment in normal and malignant cells. The aim of this study was to focus on the interaction between active species produced by 3 different types of plasma jets He, He-O_2_ and He-N_2_ and different skin cell types. Our results demonstrate that plasma treatment (He or He-N_2_) induced a significant decline in proteasome activity and a gradual cell sensitivity to CAPP.

## Results and Discussion

### Differential effects of the Helium Guided Ionization Wave (He-GIW) device on various types of human skin cells

**Figure 1:**
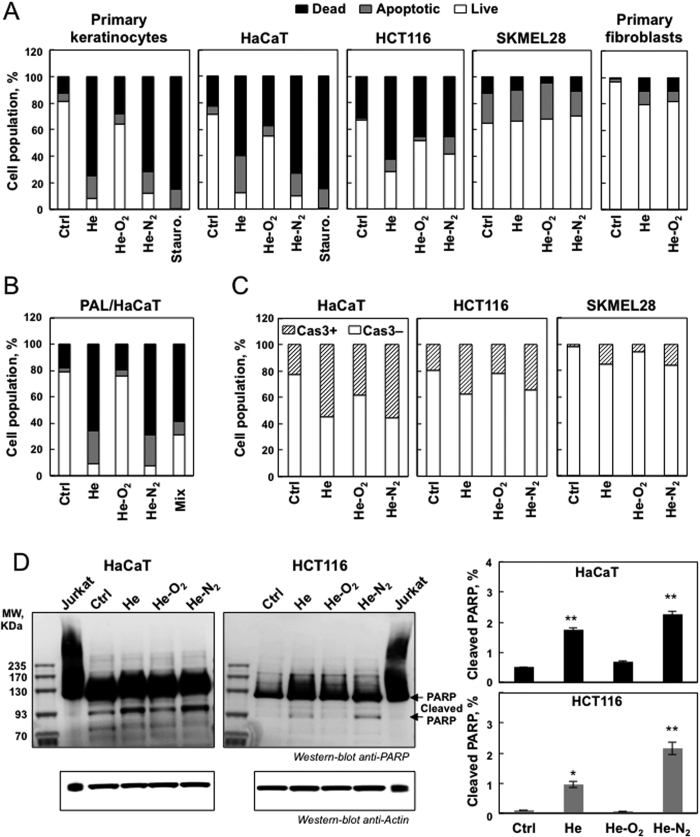
Differential apoptotic effects of CAPP on different types of human skin cells. (**A**) Primary keratinocytes, fibroblasts, HaCaT, HCT-116 and SK-MEL-28 cells were exposed to plasma treatment (He, He-O_2_ and He-N_2_) for 5 min with 1 hr post-treatment storage. Cells were stained with Annexin V-FITC and PI and analyzed by flow cytometry 24 hr after plasma treatment. Percentage of apoptotic cells (Annexin-PI positive) was shown by histogram, staurosporine treatment was used as a control of necrotic cells. The data shown is representative of three separate cultures. (**B**) Effect of plasma activated liquid (PAL) on cell viability. HaCaT cells were exposed to PAL for 1 hr (PBS treated for 5 min with He plasma, 580 μM H_2_O_2_ and 300 μM NO_2_^−^ measured in the PAL); He-O_2_ plasma (40 μM H_2_O_2_ and 50 μM NO_2_^−^ measured in the PAL) and He-N_2_ plasma (390 μM H_2_O_2_ and 300 μM NO_2_^−^ measured in the PAL) or to a mix of 580 μM hydrogen peroxide and 300 μM mM NO_2_^−^, then, stained with Annexin V-FITC and PI, and analyzed by flow cytometry 24 hr after treatment. (**C**) For the same CAPP treatment active caspase-3 was analyzed by flow cytometry. (**D**) Cleaved PARP was assessed by western blot analysis. PARP cleavage was express as a % of total PARP using Jurkat cells as a control to assess native PARP. Data, mean ± SEM from three independent cultures **P < 0.01.

Three plasmas were used in this study, all generated with the same electrical set up but using different gas mixtures (helium alone or with 1% oxygen or 1% nitrogen). We first assessed the effects of the three different CAPPs on cell viability in normal and cancerous cells. Cells were treated for 5 min with the 3 CAPPs and incubated for one hr at 37 °C. To determine whether these conditions lead to apoptosis, an Annexin/PI staining was performed 24 hr after the treatment. As shown in Fig. 1A, He and He-N_2_ CAPP induced apoptosis in human primary keratinocytes, human HaCaT keratinocytes, human colorectal carcinoma HCT-116, but very little in human primary fibroblasts, and skin melanoma-SK-MEL-28. For apoptotic cell lines a significant increase in late apoptosis (Annexin V+/PI+) was also observed (Fig. 1A) and further confirmed by the observed cleavage of caspase 3 (Fig. 1C) and its downstream effector PARP cleavage (Fig. 1D). As shown in Fig. 1A no differences in cell viability were observed between He and He-N_2_ CAPPs with treated and untreated SK-MEL-28, and a slight decrease was detected in human primary fibroblasts. Consistent with the results we did not observe cleaved caspase-3 in SK-MEL-28 cells (Fig. 1C). These results demonstrated that He and He-N_2_ CAPPs induce apoptosis in HaCaT, HCT-116 but SKMEL-28 cells seemed resistant. We confirmed several published studies showing no specific selectivity toward cancer cells^7^,^8^,^9^,^10^,^12^. Results of previous studies suggested that cancer cells which are deficient in p-53 are more sensitive to CAPP^17^. However, our results showed that p-53 cannot explain this specificity because SKMEL-28 and HCT-116 both have wild type p-53. Thus the high selectivity of CAPP toward cancer cells might be attributable to differential sensitivity of cancer cells versus primary cells to intracellular ROS or other plasma components^12^,^13^. Our study showed that there is no uniform reaction to CAPP for primary cell types, also because primary keratinocytes were very sensitive to CAPP whereas fibroblasts are not (Fig. 1A). Interestingly, many studies showing the selectivity of plasma towards cancer cells have used fibroblasts as a control and they are the most resistant to it^7^,^8^,^9^,^10^. The results obtained with He-O_2_ plasma were different from those obtained with the other two. This plasma induced little apoptosis and less cell death (Fig. 1A,C). Low levels of cleaved caspase 3 (Fig. 1C) and PARP (Fig. 1D) were observed in these cell lines after He-O_2_ plasma treatment. Moreover, this plasma induced no difference in cell viability between treated and untreated fibroblasts or SK-MEL-28 cells (Fig. 1A). Our results are in line with previous studies^13^,^17^. The addition of oxygen into the plasma gas supply made ionization extremely difficult. The plasma plume tightened very quickly leading to a decrease in the intensity of every species^18^. The addition of oxygen weakened the effect of He-CAPP on cells^11^. However, in a recent study, Joh *et al*. showed that the plasma plume properties can be adjusted by using a separated channel-mixing gas leading to efficient oxygen gas addition and enhancing the intracellular ROS production, thus improving cancer cell death^19^. Because primary keratinocytes were too sensitive to plasma treatment we decided to focus on HaCaT cells and cancer cell lines (HCT-116 and SK-MEL-28) for the rest of the study.

### Hydrogen peroxide and nitrites are the main species detected in PAL

Because a post-treatment storage time is required to induce cell death after plasma exposure and in order to gain further insight into the mechanism by which plasma treatment kills cells, we compared the efficacy of direct treatment and Plasma Activated Liquid (PAL) on HaCaT cells. We used PBS treated for 5 min with the three types of plasmas He, He-O_2_ and He-N_2_ and incubated for one hr with cells. 24 hr after treatment, for pure He and He-N_2_ plasma (Fig. 1B), we found that PAL induced the same inactivation as direct plasma exposure suggesting that the species formed in the liquid medium played a central role in the potential mechanism of cell death. For He-O_2_ plasma activated liquid (Fig. 1B), no decline in cell viability was observed indicating that the He-O_2_ mechanism of action may involve electron beam, charged particles, electric field or other short lived species and not the long-lived species present in the liquid, which indicates that this plasma is definitely very different from the other two in its mode of action.

To identify the species produced in PAL and their concentrations, PBS was treated as usual with He, He-N_2_ and He-O_2_ plasmas for 5 min. At the end of the treatment, samples were analyzed by electrochemistry at selective microelectrodes^20^. Analysis indicated that two species are produced in the liquid medium, namely H_2_O_2_ and NO_2_^−^. H_2_O_2_ produced in the PBS is much higher with pure He plasma than with He-N_2_ plasma (580 μM and 390 μM, respectively, both at 5 min) whereas concentrations in NO_2_^−^ are roughly equivalent for the two plasmas (300 μM at 5 min). In the medium treated with He-O_2_ CAPP species were not detected probably because they were present below the detection limit (200 μM) of the technique. Therefore, we used another approach, based on spectrophotometric assays, to measure these species for the He-O_2_ plasma, and measured values of 40 μM H_2_O_2_ and 50 μM NO_2_^−^ at 5 min. H_2_O_2_ and NO_2_^−^ are species known to be stable and potently toxic to cells and could be responsible for the PAL effects^4^,^12^. If these species contributed to cellular inactivation in PBS we should be able to mimic plasma treatment. As shown in Fig. 1B, H_2_O_2_ and NO_2_^−^ were added to non-plasma treated PBS at concentrations determined for 5 min of He plasma treatment (580 μM and 300 μM NO_2_^−^) and added to the HaCaT cells. The results are nearly the same as with PAL. Collectively, these findings suggest that H_2_O_2_ and NO_2_^−^ are generated in significant amounts and that they are the major RONS in He and He-N_2_ plasma treated PBS.

### Plasma treatment induced changes in cell membrane morphology and potential

**Figure 2:**
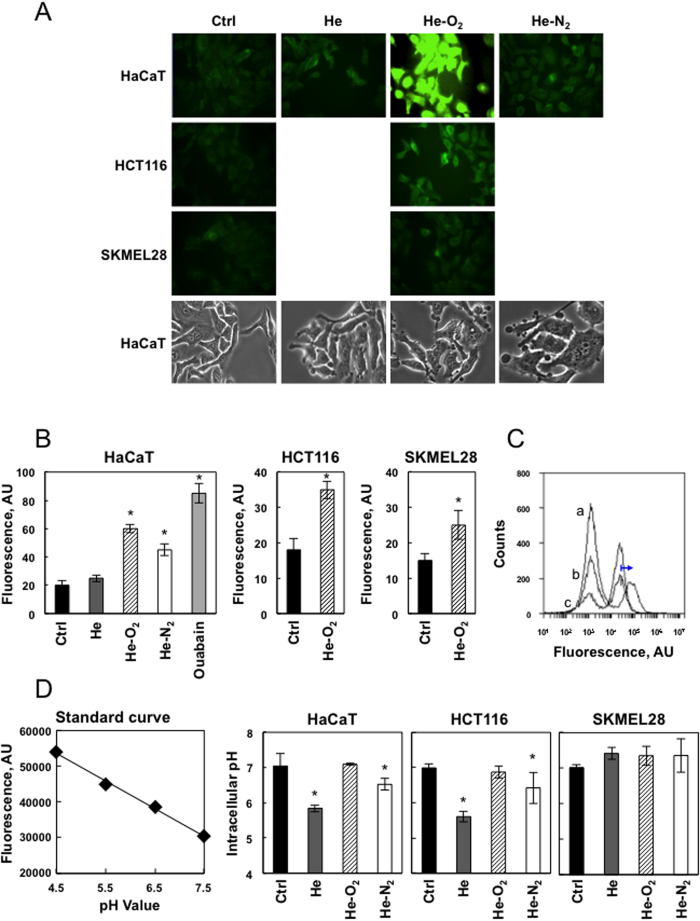
Effect of plasma treatment on membrane potential and cytosolic pH. (**A**) Cell membrane depolarization after CAPP treatment. HaCaT, HCT-116 and SK-MEL-28 cells were loaded with FluoVoltTM membrane potential dye which exhibits higher intensity with membrane depolarization, and exposed to plasma treatment (He, He-O_2_ and He-N_2_) for 5 min and imaged. Cell morphology was monitored by phase contrast microscopy after treatment. B. Data points are mean pixel intensity ± SEM (n = 10 cells) *P < 0.05. Ouabain was used as a control for total cell depolarization. (**C**) Investigation of the transmembrane potential of Hacat cells by the probe DiBAC4(3) after PAL treatment. Cells were loaded with DiBAC4(3)dye which exhibits enhanced fluorescence and a red spectral shift with membrane depolarization, and exposed to PAL treatment (He, He-O_2_) for 1 h. Cells were analyzed by flow cytometry using valinomycin which is a potassium ionophore as a control of total cell depolarization (a, valinomycin, b, PAL-He, and c, PAL- He-O_2_). (**D**) Standard curve created using pHrodo™ Green AM with Intracellular pH Calibration Buffer Kit. HaCaT cells were incubated with 10 μM pHrodo™ Green AM for 30 min at 37 °C. The Intracellular pH Calibration Buffer Kit was used to clamp the intracellular pH with extracellular buffer at pH 4.5, 5.5, 6.5 and 7.5. Intracellular pH vs. relative fluorescence units were plotted using a microplate fluorimetric reader. HaCaT, HCT-116 and SKMEL-28 cells were loaded with pHrodo™ Green AM and exposed to plasma treatment (He, He-O_2_ and He-N_2_) for 5 min and cytosolic pH was measured. Data, mean ± SEM from three independent cultures, *P < 0.05.

CAPP influenced the morphology of HaCaT cells 5 min after plasma treatment. As shown in Fig. 2A using microscopic images of phase contrast microscopy, cells changed their shape to rounded or unorganized with typical blebbing. This was more pronounced with He-O_2_ plasma treatment, an electronegative gas. We postulate that cell charging with plasma electrons and ions leads to changes in cell shape in order to minimize the electrostatic energy.

We also used the plasma membrane specific dye Fluovolt to examine cells after 5 min of plasma treatment for changes in their plasma membrane potential (PMP) at the single cell level using fluorescent microscopy (Fig. 2A). Fluovolt is a dye that responds with an increase in fluorescence intensity upon membrane depolarization. Analysis indicated that after 5 min of He, or He-N_2_ treatment, cells were depolarized and the depolarization was more pronounced for the He-O_2_ plasma. As a positive control of maximum cell depolarization, we treated cells with ouabain that collapses PMP (Fig. 2B). In contrast to the results obtained on HaCaT cells, HCT-116 and SK-MEL-28 membrane potential was less modified after He and He-O_2_ plasma treatments. We have also investigated the transmembrane potential of Hacat cells by the membrane potential probe DiBAC4(3) when cells were exposed to PBS treated for 5 min with the two types of plasmas He, He-O_2_ and incubated for one hour. The slow-response potential-sensitive probe, DiBAC4(3) can enter depolarized cells where it binds to intracellular proteins or membrane and exhibits enhanced fluorescence and a red spectral shift. As shown in Fig. 2C, cytometry analysis showed no shift in DiBAC4(3) fluorescence after PAL treatment of the cells as compared to cells treated with valinomycin which is a control of total cells depolarization.

It is well-established that cancer cells possess distinct bioelectrical properties. Notably, electrophysiological analyses in many cancer cell types have revealed a depolarized PMP that favors cell proliferation^21^. This may explain why they are less susceptible to plasma induced depolarization. Previous studies reported that plasma-induced free radicals affect the pH of the media resulting in cell death^22^,^23^. Because we used PBS as buffering agent we did not observe these changes. However, measuring intracellular pH by fluorescence spectrometry, we found that He and He-N_2_ CAPPs induced a drop of 1.2 and 0.5 pH units in cytosolic pH in HaCaT and 1.4 and 0.6 pH units in HCT-116 cells but not in SK-MEL-28 cells. Such an acidification cannot be merely explained by a the fact that CAPP triggered apoptosis^24^. Typically values drops of 0.3–0.4 pH_i_ (intracellular pH units) have been detected following exposure of cells to apoptotic stimuli. The fact that SK-MEL-28 did not show a change in pH may be due to the fact that cancer cells increase the activity and/or expression of several pH regulators, resulting in the alkalization of pH to maintain pH homeostasis and escape apoptosis^25^. He-O_2_ plasma has no effect on cellular pH (Fig. 2D). Here we report that He and He-N_2_ plasma treatment induces cellular depolarization and an early increase in intracellular protons leading to a change in intracellular ionic homeostasis that may trigger apoptosis in HaCaT cells.

### Plasma treatment compromised cellular antioxidant systems in cells

Plasma treatment results in increased rates of RONS production in the PBS. Reduced glutathione (GSH) is the first scavenger of the cell by its conversion to oxidized glutathione (GSSG). To evaluate cellular antioxidant response to plasma treatment we measured GSH. As shown in Fig. S1A, He and He-N_2_ plasma treatments induced a distinct decrease in GSH levels in HaCaT cells whereas no change in glutathione level was observed in HCT-116 and SK-MEL-28 cells (Fig. S1A). This drop in glutathione was concomitant in Nuclear Factor Erythroid 2-related factor 2 (NRF2) induction in HaCaT but not in HCT116 cells (Fig. S1B). NRF2 controls several different antioxidant pathways^26^. The first one is glutathione (GSH) production and regeneration, suggesting that cancer cells were very well equipped to present an antioxidant response whereas HaCaT cells had to induce the expression of antioxidant enzymes. As shown in Fig. S1A, He-O_2_ plasma treatment induced no changes in GSH levels in all cell types and no induction of NRF2 (Figs S1B, S1C). Using cyclic voltammetry and Amplex red analysis, we also measured the rate of hydrogen peroxide consumption by HaCaT cells after 5 min plasma treatment over one hr (Fig. S1D). The rate was the same for He and He-N_2_ plasmas but was much higher for He-O_2_ plasma. This may be due to the low level of H_2_O_2_ produced by this plasma, the level of GSH is enough to cope with RONS. However, RONS generated by the two other CAPPs exceed the scavenging capacity of antioxidant enzymes such as glutathione peroxidase which is the first H_2_O_2_ degrading enzyme in the cell leading to a decrease in the rate of converting H_2_O_2_ to water and oxygen. We also measured the rate of hydrogen peroxide consumption by HCT-116 and SK-MEL-28cells after 5 min He plasma treatment over one hr and we found that the rate were higher than for HaCaT cells that may be explain why there are more resistant to plasma treatment (Fig. S1D).

### Plasma treatment induced mild oxidative protein damage but DNA damage

**Figure 3:**
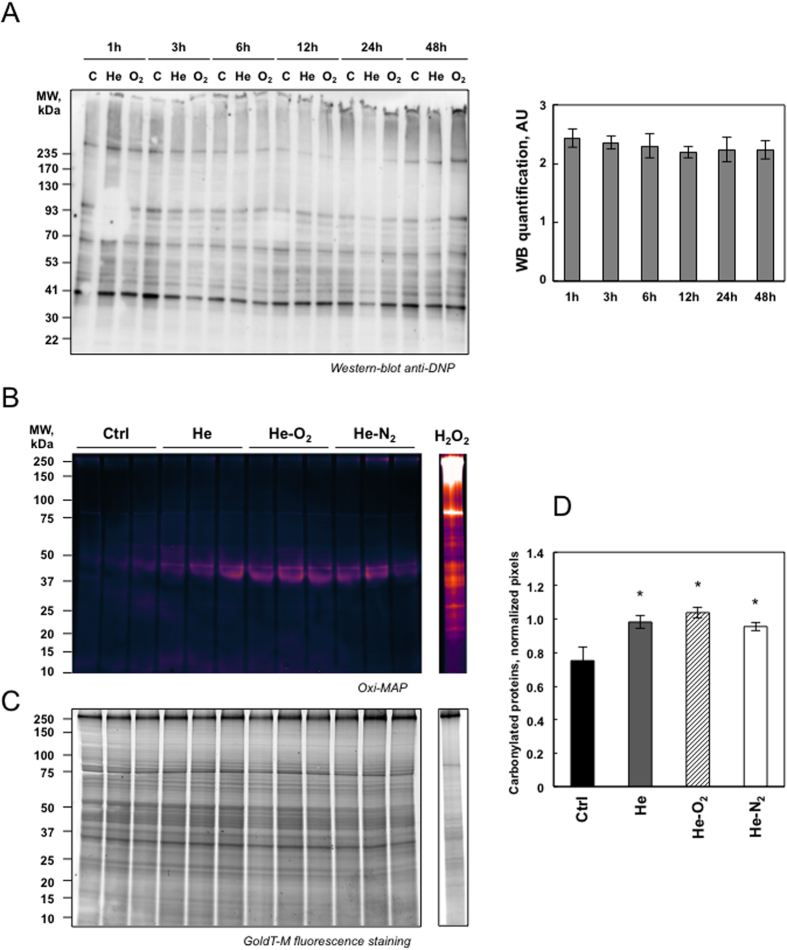
Detection of oxidatively modified proteins following plasma exposure. (**A**) HaCaT cells were exposed to plasma treatment (He, He-O_2_ and He-N_2_) for 5 min with 1 hr post-treatment storage and analyzed at different times after plasma treatment. To detect modified protein cells oxidatively, extracts were treated with 2, 4-dinitorphenylhydrazine to derivatize protein carbonyls and then evaluated by Western-blot analysis using 2, 4-dinitrophenyl antibodies and the blot were quantified using Image J. (**B**) Fluorescent detection of oxidized proteins. HaCaT cells were exposed to plasma treatment (He, He-O_2_ and He-N_2_) for 5 min with 1 hr post-treatment storage and analyzed at 24 hr after plasma treatment. Cells were lyzed and evaluated by SDS-PAGE (4–20%) pattern of carbonylated proteins pre-labeled with C5Hz. As a positive control, HaCaT cells were treated with 500 μM H_2_O_2_ for 1 hr and analyzed 24 hr later. (**C**) Total proteins post-stained with ProteinGOLDTM. (**D**) Semi-quantification of carbonylated proteins were performed by densitometric analysis, expressed as relative values (normalization to total protein) and shown as mean ± S.D (n = 3) and analyzed using Student’s t-test; *P < 0.05.

**Figure 4:**
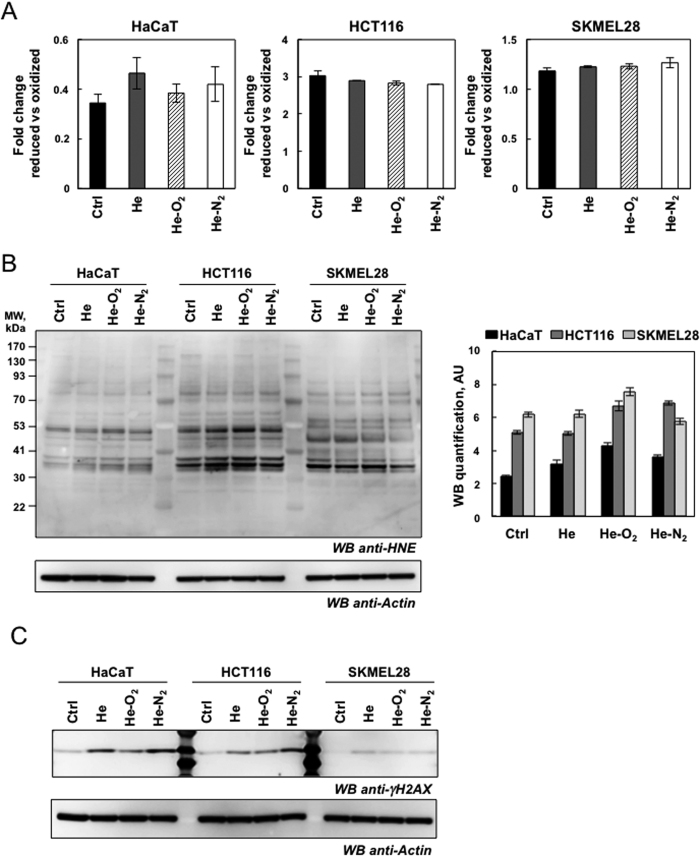
Detection of lipid peroxidation and DNA damage following plasma exposure. (**A**) Lipid peroxidation detection with Image-iT^®^ Peroxidation Kit. HaCaT, HCT-116 and SK-MEL-28 cells were stained with 10 μM Lipid Peroxidation Sensor for 30 min and exposed to plasma treatment (He, He-O_2_ and He-N_2_) for 5 min with 1 hr post-treatment and analyzed after plasma treatment with a microplate fluorimetric reader. In control cells, most of the signal is in the red channel and the ratio of 590/510 is high, data, mean ± SEM from three independent cultures (**B**) HaCaT, HCT-116 and SKMEL-28 cells were exposed to plasma treatment (He, He-O_2_ and He-N_2_) for 5 min with 1 hr post-treatment and analyzed 24hr after plasma treatment. Lipid peroxidation was detected by immunoblotting against 4-hydroxy-nonenal using whole-cell lysates (*n* = 3). Actin was used as loading control for quantification. (**C**) For the same CAPP treatment DNA damage was evaluated by western blot analysis using polyclonal antibody against Phospho-Histone H2AX, whole-cell lysates.

Plasma treatment resulted in increased rates of RONS. Thus, using immunological detection of protein carbonyls, we sought information on relative alterations in the levels of oxidized proteins due to plasma treatment. Carbonyl functional groups can be introduced into proteins by a variety of oxidative processes including direct oxidation of amino acid with H_2_O_2_ or reaction of lipid peroxidation products from cellular membrane oxidation. As shown in Fig. 3A, plasma treatment of HaCaT cells with the two types of devices induced no distinct increase in the levels of oxidatively modified protein even at different times after plasma exposure (one to 48 hr after 5 min plasma treatment and one hr storage in PBS). In order to be more sensitive we used a fluorescent Oxi-map method and compared the effect of the three devices. As shown in Fig. 3B and D oxidative modification was not global in nature but appeared specific to distinct proteins with molecular weight of 42–45 kDa which may be actin, some of which exhibited a greater degree of oxidation than others. Our results are in line with a previous study showing that protein oxidation due to CAPP treatment only targeted certain proteins and mainly actin^16^. It has been previously shown that oxidative modification of actin can cause alteration in cell cytoskeletal proteins and activate apoptotic pathways and changes in cell morphology^27^. Therefore the change in cellular shape after plasma treatment may be due to the oxidation of actin (Fig. 2A). Another protein around 83 kDa may be carbonylated aconitase. A general deficit of Fe-S cluster proteins, such as aconitase, a tricarboxylic acid cycle mitochondrial enzyme that contains a 4F–4S cluster as a prosthetic group, is a phenotype classically associated with oxidative stress in the mitochondria^28^. To evaluate the effect to plasma treatment on aconitase activity, we isolated mitochondria from HaCaT cells 24 hr post-treatment and measured the activity of the enzyme (Fig. S2A). Aconitase was inactivated, especially after He and He-N_2_ plasma treatments. As α-ketoglutaratedehydrogenase (KGDH) has been described as an oxidation target in various biological systems we measured its activity^29^. As shown in Fig. S2B, KGDH was mainly inactivated after He and He-N_2_ plasma treatments. We found that these two proteins which are Krebs cycle enzymes, which are largely responsible for respiration and energy metabolism were oxidized and that CAPP treatment seemed to target only specific proteins. Surprisingly, the level of protein oxidation was higher and targeted all proteins when HaCaT cells were treated with 500 μM H_2_O_2_ (Fig. 3B). We also measured total lipid peroxidation production in HaCaT, HCT-116 and SK-MEL-28 cells 24 hr later (5 min plasma treatment, one hr storage). As shown in Fig. 4A, we detected no increase in lipid peroxidation after exposure of the cell to the three devices and in all cell types. 4-Hydroxy-2-nonenal (HNE), an α, β unsaturated aldehyde is a major product of lipid peroxidation and very toxic^30^. Utilizing antibodies specific to HNE-Michael adducts we detected no increase in the HNE content of protein after plasma treatment compared to control cells (Fig. 4B) suggesting that changes in cell morphology are not due to peroxidation, and oxidation of the membrane lipid and may be due to a depolarization of the cell by an electrical field^31^,^32^. To determine if CAPP treatment could induce DNA damage, we looked at phosphorylation of H2AX which is used to quantify accumulation of DNA damage. Western blot against H2AX phosphorylated at Ser-139 revealed that He and He-N_2_ plasma treatment of cells induced an increase in DNA damage in HaCaT and HCT116, the effects were more pronounced with He-N_2_ plasma and He-O_2_ induced mild DNA damage (Fig. 4C). However the three plasma created the same level of DNA in SK-MEL-28 even if these cells are very efficient at repairing DNA damage^33^.

### Plasma treatment induced alterations in proteasome peptidase activity

**Figure 5:**
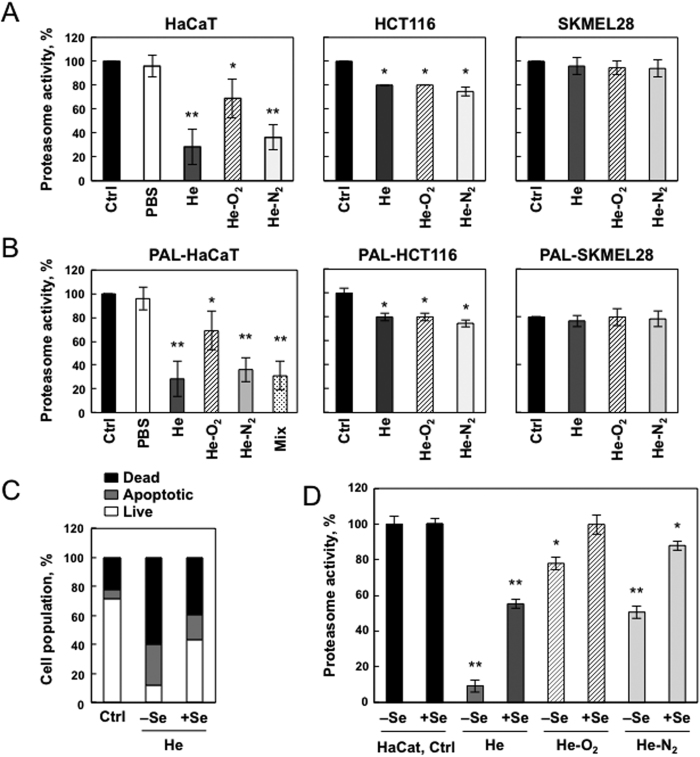
Proteasome inactivation following plasma exposure. (**A**) HaCaT, HCT-116 and SK-MEL-28 cells were exposed to plasma treatment (He, He-O_2_ and He-N_2_) for 5 min with 1 hr post-treatment storage. Proteasome chymotrypsin-like activity was measured 24 hr post treatment using the fluorogenic peptide LLVY-AMC. Proteasome activity is presented as a percent of non-treated cells. Data, mean ± SEM from three independent cultures, *P < 0.05; **P < 0.01. (**B**) Effect of plasma activated liquid (PAL) on cell proteasome activity. HaCaT, HCT-116 and SK-MEL-28 cells were exposed to PAL for 1 hr (PBS treated for 5 min with He plasma (580 μM H_2_O_2_ and 300 μM NO_2_^−^ measured in the PAL); He-O_2_ plasma (40 μM H_2_O_2_ and 50 μM NO_2_^−^ measured in the PAL) and He-N_2_ plasma (390 μM H_2_O_2_ and 300 μM NO_2_^−^ measured in the PAL) or to a mix of 580 μM hydrogen peroxide and 300 μM mM NO_2_^−^ for HaCAT cells and proteasome activity was measured 24 hr post treatment. Data, mean ± SEM from three independent cultures, *P < 0.05; **P < 0.01. (**C**) Effect of glutathione peroxidase overexpression on apoptosis following plasma treatment. HaCaT cells were grown in medium depleted or supplemented in Selenium (Se), a condition which is known to increase glutathione peroxidase activity and were exposed to He-plasma treatment for 5 min with 1 hr post-treatment storage. Cells were stained with Annexin V-FITC and PI and analyzed by flow cytometry 24 hr after plasma treatment. Percentage of apoptotic cells (Annexin-PI positive) was shown by histogram. (**D**) Effect of glutathione peroxidase activation on proteasome activity following plasma treatment. HaCaT cells were grown in medium depleted or supplemented in Selenium (Se), a condition which is known to increase glutathione peroxidase activity and expression and then exposed to CAPP treatment (He, He-O_2_ and He-N_2_) for 5 min with 1 hr post-treatment storage. Proteasome activity was measured 24 hr post treatment. Data, mean ± SEM from three independent cultures, *P < 0.05; **P < 0.01.

The ubiquitin-proteasome system is an important regulator of cell growth and apoptosis^14^. CAPPs produce RONS that lead to the production of oxidized proteins which are preferentially degraded by the proteasome. Thus the effects of 3 different types of plasma jet treatments, He, He-O_2_ and He-N_2_, on proteasome activity were evaluated 24 hr after 5 min plasma treatment and one hr storage in PBS, in HaCaT, HCT-116 and SK-MEL-28 cells. As shown in Fig. 5A proteasome was inactivated in HaCaT cells after the 3 different types of plasma jet treatments, but proteasome was more sensitive after He and He-N_2_ exposure than He-O_2_ CAPP (75% and 40% inactivation, respectively). In HCT-116 proteasome inactivation was 20% for the three plasmas (Fig. 5A). In SK-MEL-28 cells, proteasome activity did not vary between control and plasma treated cells indicating that these cells are very well protected against oxidative injury. Furthermore, declines in proteasome activity were not due to a loss of proteasome content as judged by label-free quantitative proteomic analysis (Table S1). We were able to reproduce these data using PAL (PBS treated for 5 min with the three types of plasma He, He-O_2_ and He-N_2_) and incubated for one hr with cells, indicating that the observed alterations in proteasome function were due to events which occurred in the plasma-treated liquid (Fig. 5B). As shown in Fig. 5B, H_2_O_2_ and NO_2_^−^ were added to non-plasma treated PBS at concentrations determined for 5 min of He plasma treatment (580 μM and 300 μM NO_2_^−^) and added to the HaCaT cells, proteasome was inhibited to the same extent than with PAL. Glutathione peroxidase (GPx1) is a selenium containing enzyme in charge of hydrogen peroxide detoxification in cells. Supplementation of selenium in the cell media resulted in a 40% increase in GPx1 expression and activity^34^. We found that cells that overexpress GPx1 are protected from apoptosis induced by He-plasma treatment (Fig. 5C). Moreover, as shown in Fig. S1D, the rate of hydrogen peroxide consumption in cells supplemented with selenium and treated for 5 min by plasma was higher. We found that proteasome activity was protected in cells that overexpressed glutathione peroxidase showing that proteasome inactivation was mainly due to hydrogen peroxide (Fig. 5D). We cannot also rule out that proteasome subunits may get oxidized by hydrogen peroxide.

### Plasma treatment induced alterations in mitochondrial transmembrane potential

It is well known that proteasome dysfunction is a consequence of oxidative stress and that proteasome inhibition induces mitochondrial dysfunction^35^,^36^,^37^.

**Figure 6:**
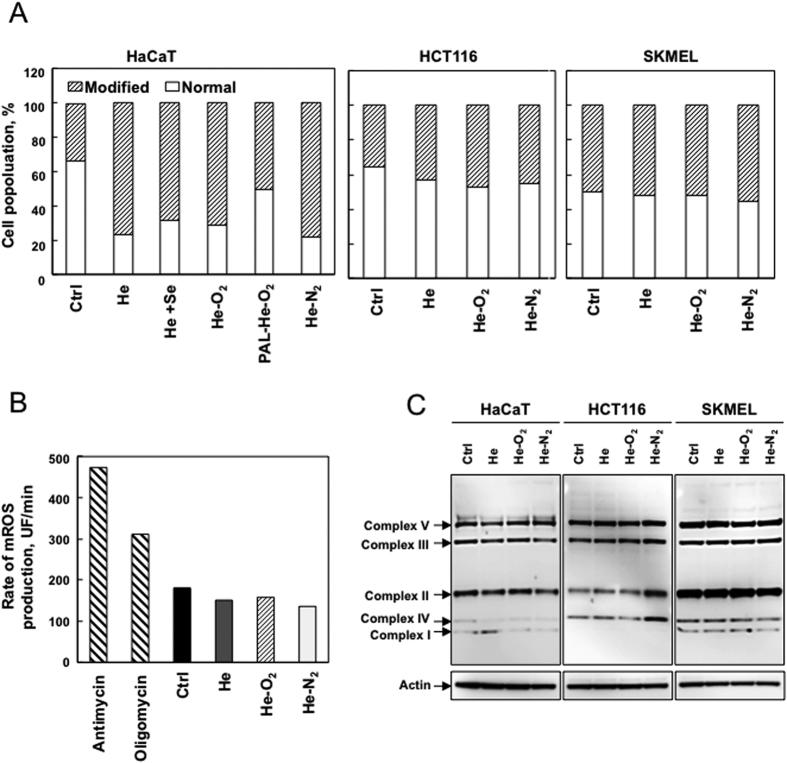
Collapse of the mitochondrial transmembrane potential without ROS production following plasma treatment. (**A**) HaCaT cells were grown in normal or medium supplemented in Selenium (Se), a condition which is known to increase glutathione peroxidase activity and expression, HCT-116 and SK-MEL-28 cells were exposed to plasma treatment (He, He-O_2_ and He-N_2_) for 5 min with 1 hr post-treatment storage. Hacat cells were also exposed to PAL for 1 hr, PBS treated for 5 min with He-O_2_ plasma (40 μM H_2_O_2_ and 50 μM NO_2_^−^ measured in the PAL). Mitochondrial membrane potential was measured using JC-1 by flow cytometry 24 hr post treatment and expressed as a percent of cells with a normal membrane potential. The data shown is representative of three separate cultures. (**B**) For the same CAPP treatment, rate of superoxide production was measured in HaCaT cells by flow cytometry using MitoSox. Antimycin (4 μg/ml) and oligomycin (1 μg/ml) induced a significant increase in mitochondrial ROS (mROS) production and were used as positive controls. (**C**) Immunoblot analysis of OXPHOS complexes (CI to CV) protein levels in cells following the same CAPP treatment, with actin as a loading control. The data shown is representative of three separate cultures.

Therefore, we sought further information regarding mitochondrial function. We subjected plasma treated HaCaT, HCT-116 and SK-MEL-28 cells to the JC-1 cationic dye in a FACS analysis to examine whether the three devices resulted in a drop in mitochondrial membrane potential (MMP). As shown in Fig. 6A, only HaCaT cells demonstrated a significant decrease in JC-1 red-green fluorescence following treatment with all the three plasmas: He, He-O_2_ and He-N_2_. In contrast, plasma treatment of cancer cells did not change their MMP. Surexpression of glutathione peroxidase in Hacat cells exposed to He-CAPP resulted in less depolarization of mitochondria suggesting that RONS present in the liquid may be involved. To see whether this collapse could induce mitochondrial ROS production, we measured mitochondrial superoxide using Mitosox, a fluorogenic probe for the specific detection of superoxide in the mitochondria of HaCaT cells. We did not detect an increase in mitochondrial ROS production after plasma treatment (Fig. 6B). We used antimycin and oligomycin treated cells as controls, conditions known to induce mitochondrial ROS production. As shown in Fig. 6C, the steady-state amount of several components of the OXPHOS complexes, including subunits of the respiratory complexes I, II, III and IV, were slightly changed after plasma treatment in all cell types. Our results are in line with previous studies showing that the increase of plasma-induced ROS can occur through the mitochondria-independent cellular response^38^. The fact that He-O_2_ CAPP is decreasing cellular MMP to the same extent as the two others plasmas indicates that RONS which are produced in very low amounts in the liquid by this CAPP are likely not responsible for mitochondrial depolarization. Moreover, PBS treated for 5 min with He-O_2_ CAPP prevents the drop in mitochondrial membrane potential. The inactivation of cancer cells by electrochemical means has been well characterized^39^ and involves different mechanisms such as changes in the plasma membrane potential. These changes in plasma membrane potential and MMP may lead to local ion flux imbalances^39^,^40^,^41^,^42^. Full characterization of the electric field generated by our three devices and mainly He-O_2_ CAPP need to be performed in order to understand the drop in MMP.

### Plasma treatment targets only a subset of the total proteome involved in key cellular pathways

**Figure 7:**
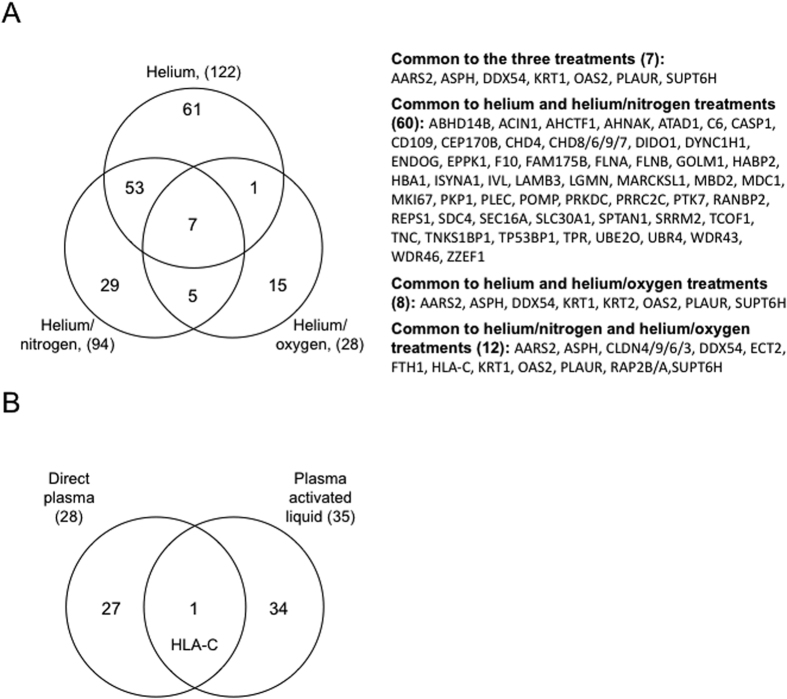
Venn diagram of proteins of varying abundance after various plasma treatments. (**A**) HaCaT cells were exposed to plasma treatment (He, He-O_2_ and He-N_2_) for 5 min with 1 hr post-treatment storage and analyzed at 24 hr after plasma treatment. Total protein extracts were analyzed nanoLC–MS/MS after trypsin digestion. Differential label-free quantitative analyses between treatment and control were performed. (**B**) Venn diagram of proteins of varying abundance after direct plasma or plasma activated liquid treatments. HaCaT cells were exposed to He-O_2_ plasma for 5 min with 1 hr post-treatment storage or PAL for 1 hr (PBS treated for 5 min by He-O_2_ plasma) and analyzed at 24 hr after plasma treatment. Total protein extracts were analyzed by nanoLC–MS/MS after trypsin digestion. Differential label-free quantitative analyses between treatment and control were performed.

Although many cellular pathways have been identified as a response to plasma treatment, in most cases the protein targets have not been identified^43^. The identification of these proteins may give insight into the mechanisms by which plasma treatment could affect cellular function. For this purpose, proteins derived from total cellular extracts of HaCaT cells exposed to plasma treatment (He, He-O_2_ and He-N_2_) for 5 min, 1 hr post-treatment storage and 24 hr post treatment were analyzed using label-free quantitative proteomics. For each sample, 3 cell cultures were prepared in parallel and total cell extracts were analyzed by nanoLC–MS/MS after trypsin digestion. In total for all conditions analyzed more than 3600 proteins were identified and quantified. Differential quantitative analysis between each plasma treatment and control condition led to 122, 94, and 28 varying proteins for He, He-N2, and He-O2, respectively (Fig. 7, Table S1). The identified proteins with varying intensities between plasma treatment and control were analyzed for known function and grouped by functional correlation, biological pathways and interaction analysis by Ingenuity (IPA) (Table 1). Major molecular functions including several aspects of carbohydrates, amino acids, lipids and nucleic acids metabolism, cellular morphology and assembly, cellular function and maintenance, protein degradation, cell growth and proliferation are all affected by plasma treatment, which may explain why cells triggered apoptosis. Venn diagrams were constructed to identify common and specific differential expressed proteins to plasma exposure (Fig. 7A). Our results showed that He, and He-N_2_ which appeared to have the same toxicological effects on cells and produce the same amount of RONS in the liquid have 50% of proteins in common. Seven proteins are common to the three plasmas and our results confirmed that He-O_2_ is very different form the two others. It contains only 12 proteins in common with He-N_2_ and 8 with He plasma.

We also performed proteomic analysis on protein extracts from cells treated by PAL (PBS treated for 5 min with He-O_2_ and incubated for one hr with cells) 24 hr after the treatment. We found only one protein in common with direct plasma treated cells (Fig. 7B) suggesting that for this plasma, RONS produced in the liquid are not only responsible for the cellular response but that the electric field and others plasma components may be involved^31^,^32^. IPA analysis revealed that the main cellular pathways affected by plasma treatment were cell adhesion, response to stress and infection, and extracellular matrix receptor interaction. We did not detect a NRF2 mediated oxidative stress response as has been described in a previous study^43^ because we only looked at 24 hr after plasma treatment. However, our data are in line with their gene expression profiling reports obtained 24 hr after plasma treatment^43^. We found that the same pathways were affected by plasma^44^. Extracellular matrix (ECM), a complex network of macromolecules with distinctive physical, biochemical, and biomechanical properties, is the most targeted pathway by He and He-N_2_ plasmas. This indicates that they may be harmful to normal cells and could be associated with cancer formation. Interestingly, some studies have shown that ECM is commonly deregulated and becomes disorganized in diseases such as cancer^45^. We also found that many proteins involved in cell adhesion and cellular component organization are also modified in their expression. These data are in line with the shape of the cells after plasma treatment (Fig. 2A). This indicates that loss of cell adhesion to ECM was already significant prior to cell death and that CAPP facilitated cell death may be related to integrin-ECM interactions.

## Conclusion

In summary, our results provide support for the hypothesis that upon treatment with He and He-N_2_, free radicals generated in the liquid mediate apoptosis due to DNA damage, mitochondrial potential collapse and proteasome inactivation in HaCaT cells. He-O_2_ plasma effect is very mild, it induces cell depolarization that can be related to the electric field associated with the ionization front or generated in the plasma environment. Ongoing efforts to identify species produced in the liquid, and the impact of plasma generated electric field will enable the elucidation of proteasome mechanism of inactivation in order to modify the plasma device so they can be more selective to cancer cells.

## Methods

### Chemicals and antibodies

All chemicals were purchased from Sigma-Aldrich (Saint Quentin Falavier, France). Phosphate buffered saline, PBS (1.5 mM KH_2_PO_4_, 155 mM NaCl, 2.70 mM Na_2_HPO_4_^−^7H_2_O, pH 7.2) was used in this study.

### Cell culture

HaCaT keratinocyte cells were obtained from Thermofischer (Saint Aubin, France). Primary cultures of human dermal keratinocytes were obtained from M. Moreau (Inserm U505, France) and were grown in Epilife medium (Thermofischer, Saint Aubin, France). Primary cultures of human dermal fibroblasts were obtained from Dr Valérie Planat (Stromalab, Université Paul Sabatier, Toulouse, France). HCT-116 and SK-MEL-28 were obtained from Dr Pascal Descargues (Genoskin, Toulouse, France) and were grown in Dulbecco’s modified Eagle’s medium supplemented with 10% fetal calf serum. For selenium supplementation, HaCaT cells were grown and maintained as described elsewhere^34^.

### The Helium Guided Ionization Wave (He-GIW) device

The plasma process consists of the production of guided ionization waves at atmospheric pressure and room temperature. This has been previously characterized in other studies^18^,^46^,^47^,^48^,^49^. Process gas was either He, He/1% N_2_ mixture or He/1% O_2_ mixture at a 2 standard liters per minute (slm) flow rate. Plasma was generated by applying a 7.5 kV, 10 kHz, 1% duty cycle, with a positive nanosecond pulsed wave potential between the two electrodes^18^,^47^.

### Plasma treatment

Three different plasma mixtures were used: He, He-1% O_2_ and He-1% N_2_. For each condition, cells at 80% confluency were treated for 5 min in a 6-well plate containing 2 mL PBS. The distance between the sample surface and the output of the reactor was fixed at 15 mm. Cells were incubated for 1 hr at 37 °C after plasma treatment, PBS was removed and cellular analyses were carried out 24 hr later.

### Plasma activated liquid (PAL) effect on cells

PBS was treated for 5 min with He, He-1% N_2_ or He-1% O_2_ plasmas. Immediately, the treated liquid was incubated with cells for 1 hr then replaced with culture medium.

### Detection and identification of the main species produced in PBS after He and He-N_2_ plasma treatment

The nature and the concentrations of stable chemical species produced in PBS after plasma treatment, were measured using an electrochemical method named cyclic voltammetry at platinzed microelectrodes^20^,^50^.

### Determination of hydrogen peroxide and nitrite

H_2_O_2_ levels generated by He-O_2_ plasma treatment were measured using the Amplex Red assay following the manufacturer’s protocol (Thermofischer, Saint Aubin, France). Calibration curve was performed using hydrogen peroxide concentrations varying from 5 to 100 μM in PBS. Levels of nitrite NO_2_^−^ were determined using the Griess assay according to the manufacturer’s protocol (Thermofischer, Saint Aubin, France).

### Flow cytometry analysis of apoptosis

Annexin V-FITC/PI apoptosis detection kit was used as described by the manufacturer (Thermofischer, Saint Aubin, France). Flow cytometric analysis (BD Accuri™ C6 flow cytometer, BD Biosciences, Le Pont de Claix, France) of apoptotic populations, positive for active-capsase-3 was carried out using the PE-Active caspase-3 apoptosis kit (Thermofischer, Saint Aubin, France).

### Measurement of acute changes in plasma membrane potential

Acute changes in the plasma membrane potential were measured by fluorescent microscopy using fluorescent dye that are sensitive to membrane potential FluoVolt^TM^ and DiBAC4(3) (Thermofischer, Saint Aubin, France) as described by the manufacturer. To assess the effects of total membrane disruption oubain 10 nM or 10 μM valinomycin was added to the cell culture medium. MATLAB software was used to calculate pixel intensities.

### Measurement of intra-cellular pH

Cytosolic pH after CAPP treatment was measured using pHrodo™ Green AM with an Intracellular pH Calibration Buffer Kit (Thermofischer, Saint Aubin, France). Briefly, cells were incubated with 10 μM pHrodo™ Green AM for 30 min at 37 °C. A standard pH curve was produced using different buffer solutions. Intracellular pH vs. relative fluorescence units was plotted using a microplate fluorimetric reader (BMG-FLUOstar Galaxy, Stuttgart, Germany), excitation/emission wavelengths were 509/533 nm.

### Lipid peroxidation analysis

Visualization of lipid peroxidation *in-situ* was carried out through labeling cells with C11-BODIPY581/591, a fatty acid analogue that readily incorporates into cell membranes and whose fluorescence irreversibly changes from red to green upon exposure to ROS (Image it, Thermofischer, Saint Aubin, France). Samples were then analyzed at 37 °C using a microplate fluorimetric reader (BMG-FLUOstar Galaxy, Stuttgart, Germany). Red emission from intact C11-BODIPY581/591 was detected at 580–620 nm and green emission that indicated peroxidation at 495–560 nm.

### Determination of cytosolic glutathione levels (GSH)

Glutathione levels were determined using Thiol tracker violet detection reagent ((Thermofischer, Saint Aubin, France) and analyzed using a microplate fluorimetric reader (BMG-FLUOstar Galaxy, Stuttgart, Germany), excitation/emission wavelengths were 405/526 nm.

### Mitochondrial membrane potential

Mitochondrial inner membrane potential was measured as previously described^51^. using JC-1 probe (Thermofischer, Saint Aubin, France).

### Mitochondrial ROS production

Cells were trypsinized and counted using BD Accuri™ C6 flow cytometer (BD Biosciences, Le Pont de Claix, France). Cells were incubated with 5 μM MitoSOX™ probe (Thermofischer, Saint Aubin, France) for 5 min at room temperature, and incubated with different media: standard medium, with oligomycin (2 μg/mL) or antimycin (4 μg/mL). Fluorescent kinetics were recorded in the cytometer for10 min.

### Assay for aconitase activity

Mitochondria were isolated as previously described^51^. Mitochondria were suspended in 25 mM phosphate buffer pH 7.25 supplemented with 0.05% Triton X-100 and aconitase activity was assayed spectrophotometrically at 340 nm, as previously described^52^.

### Assay for KGDH

Mitochondria were diluted in 25 mM MOPS, 0.05% Triton X-100, pH 7.4, and KGDH activity was assayed spectrophotometrically as the rate of NAD^+^ reduction to NADH as previously described^35^.

### Proteasome peptidase activity

Peptidase activity of the proteasome was assayed using a fluorogenic peptide, succinyl-Leu-Leu-Val-Tyr-7-Amido-4-Methylcoumarin (LLVY-AMC) Sigma-Aldrich (Saint Quentin Falavier, France) as previously described^36^.

### Western blot analysis

Cellular lysis was performed using a lysis buffer (1.5 mmol/L EDTA, 50 mmol/L Hepes pH 7.4, 150 mmol/L NaCl, 10% (v/v) glycerol, and 1% (v/v) NP40). Total cellular lysates were loaded onto a 4% to 20% SDS-PAGE gel (Bio-Rad), transferred onto nitrocellulose membrane, and revealed with different antibodies such as homemade anti-proteasome^36^ and anti-HNE 4-hydroxy-nonenal^37^. Commercial antibodies used were, MitoProfile^®^ Total OxPhOS WB Antibody Cocktail and anti-phospho-gamma H2AX-Ser139 (Thermofischer, Saint Aubin, France), anti-actin and anti-NRF2 (Abcam, Paris, France) and anti-human PARP (BD Biosciences, Le Pont de Claix, France). Direct recording of the chemi-luminescence (GeneGnome Syngene) and quantification (GeneSnap software) were performed (Ozyme, St Quentin en Yvelines, France).

### Detection of carbonylated proteins

Carbonylated proteins were detected and analyzed after the derivatization of protein carbonyl groups with 2,4-dinitrophenylhydrazine (DNPH) (Protein Oxidation Detection Kit, OxyBlot™, Millipore, Molsheim, France).

For Oxi-map analysis (Oxiproteomics, Paris, France), carbonylated proteins were labeled with CyDyeTM hydrazides (GE Healthcare) as described previously^53^,^54^. Carbonylated proteins were labeled with Cy5 hydrazides (GE Healthcare) and total proteins were precipitated and resuspended in loading buffer and separated by SDS-PAGE (4–20%). Total proteins were post-stained with ProteinGOLD (Gel company). Fluorescent scanning was performed using the Ettan Dalt system (GE Healthcare) at excitation and emission wavelengths of 635/680 nm for the Cy5 hydrazide and 390/595 nm for total proteins, respectively.

### Label-free quantitative proteomics analysis

Reduction and alkylation of cysteine residues were performed by diluting 50 μg of each sample in Laemmli buffer for 5 min at 95 °C followed by a treatment with 90 mM chloroacetamide for 30 min at room temperature in the dark. The samples were loaded on a SDS-PAGE gel and proteins were concentrated in one band. Proteins were in-gel digested overnight with a solution of modified trypsin (20 ng/μl, sequence grade, Promega, Charbonnières, France) at 37 °C. The resulting peptides were extracted from the gel and dissolved in 125 μl of 2% acetonitrile (ACN), 0.05% trifluoroacetic acid (TFA). Three independent nano-LC–MS/MS analyses were performed for each sample using an Ultimate 3000 NRS system (Dionex, Amsterdam, The Netherlands) coupled to an Orbitrap Q-Exactive Plus mass spectrometer (Thermo Fisher Scientific, Bremen, Germany). The Orbitrap Q-Exactive Plus was operated in data-dependent acquisition mode with the XCalibur software. Survey scans MS were acquired in the Orbitrap on the 150–1500 *m/z* range with the resolution set to 70,000.

The Mascot Daemon software (Version 2.4, Mascot server 2.4, Matrix Science, London, UK) was used to perform searches in human taxonomy using the SwissProt database (UniProt release 2014_04). Mascot results were parsed with the in-house developed software, Mascot File Parsing and Quantification (MFPaQ, version 4.0.0)^55^ based on the target-decoy strategy, FDR peptide level was set at 1% by adjusting peptide p-value threshold. Quantification of proteins was performed using the label-free module implemented in the MaxQuant software (version 1.5.0.1). The ratios O_2_-Ctrl, He-Ctrl or N_2_-Ctrl were determined by the sum of intensity values in three biological replicate analyses. For differential conditions, a Student *t*‐test on the normalized intensity values was used for statistical evaluation of the significance of expression level variations. A 2-fold change and a p-value of 0.05 were used as combined thresholds to define biologically regulated proteins. To obtain information on protein expression pattern Ingenuity pathway analysis (Ingenuity systems, Mountain View, CA, USA) and String analysis were carried out.

### Statistical analysis

Results were expressed as mean ± SEM and analyzed using GraphPad Prism 5 Software. The Mann–Whitney and one-way ANOVA tests were used to compare data sets. Statistical significance was set at *P* < 0.05.

## Additional Information

**How to cite this article**: Dezest, M. *et al*. Mechanistic insights into the impact of Cold Atmospheric Pressure Plasma on human epithelial cell lines. *Sci. Rep.* **7**, 41163; doi: 10.1038/srep41163 (2017).

**Publisher's note:** Springer Nature remains neutral with regard to jurisdictional claims in published maps and institutional affiliations.

## Supplementary Material

Supplementary Data

## Figures and Tables

**Table 1:** Ingenuity and string pathway analysis of the top molecular and cellular functions for the action of plasma in HaCaT cells.

Biological process	GO number	fold enrich.	*p* value	Molecules
**Helium plasma**				
hemidesmosome assembly	31581	58.30	6.47E-03	LAMC2, PLEC, LAMB3, LAMA3
cellular component organization	16043	1.90	1.32E-03	AARS2, LRSAM1, LAMC2, MKI67, AP2A2, ASUN, PLEC, CCNB2, FLNA, SEC16A, SRP72, TPR, HBA1, HBA1, THBS1, WDR43, PTK7, POMP, LAMB3, MBD2, CD44, AHCTF1, APOB, ENDOG, VTN, ACIN1, GOLM1, ATAD1, EHD3, PRPF8
nucleus organization	6997	10.93	6.81E-03	ASUN, CCNB2, TPR, AHCTF1, GOLM1, SYNE2, POLR1B, RANBP2
response to abiotic stimulus	9628	3.13	4.43E-02	MKI67, FAM175B, TPR, THBS1, APOB, ENDOG, SERPINB13, LGMN, APP, SDC4, CASP1, HMGN1, MAP2K4, STK39, RANBP2, APOM, IVL, PRKDC, TP53BP1, FANCD2
response to stress	6950	2.11	2.82E-03	MKI67, FAM175B, FLNA, TPR, HBA1, HBA1, THBS1, PTK7, CD44, F10, APOB, OAS2, ENDOG, VTN, ACIN1, POLR2G, EHD3, LGMN, APP, SDC4, DHX36, DDX58, CASP1, HLA-DRA, UFC1, ASS1, HMGN1, STK39, MAP2K4, PYCARD
**Helium/nitrogen plasma**				
response to stress	6950	2.26	4.78E-03	CDK7, LYN, MKI67, ECT2, FAM175B, FLNA, ID1, IFIT1, TPR, HBA1, HBA1, PTK7, F10, TMEM109, OAS2, ENDOG, ACIN1, LGMN, SDC4, CASP1, TRIM21, NUP214, MX1, RIF1, APOBEC3B, PLAUR, RANBP2, SLC30A1, PLEK, SPTAN1
cellular component organization or biogenesis	71840	2.03	1.02E-03	AARS2, LYN, MKI67, ECT2, PLEC, FLNA, SEC16A, ID1, TPR, HBA1, RRP1, HBA1, WDR43, PTK7, POMP, SCAF11, LAMB3, MBD2, FTH1, AHCTF1, ENDOG, WDR46, ACIN1, GOLM1, ATAD1, APLP2, SDC4, PKP1, TRIM21, HDAC7
**Cellular components**	**GO number**	**fold enrich.**	***p*** **value**	**Molecules**
**Helium plasma**				
laminin complex	43256	65.59	1.78E-02	LAMC2, LAMB3, LAMA3
focal adhesion	5925	5.94	4.17E-04	SYNE2, AHNAK, FLNB, IGF2R, PLAUR, TNC, MDC1
nucleolus	5730	3.38	2.50E-02	DDX54, MKI67, FLNA, SRP72, WDR43, WDR46, ACIN1, TCOF1, POLR2G, POLR1B, RABL6, ABHD14B, FTSJ3, PYCARD, PRKDC, SRP19
extracellular membrane-bounded organelle	65010	2.57	8.65E-06	SERPINB3, PLEC, FLNA, ABCB6, HBA1, HBA1, THBS1, CD44, AHCTF1, APOB, VTN, GOLM1, SERPINB13, DCTD, LGMN, APP, SDC4, DHX36, PKP1, SYNE2, HLA-DRA, UFC1, ASS1, AHNAK, FLNB, IGF2R, ABHD14B, FBP1, PLAUR, LAMA3
cytoskeleton	5856	2.35	3.69E-02	CCNB2, PLEC, FLNA, TPR, WDR43, APOB, APP, DDX58, PKP1, SYNE2, RABL6, EPPK1, AHNAK, DIDO1, CEP170B, FLNB, STK39, UBR4, DYNC1H1, SPTAN1, CEP164, TNKS1BP1, KRT1, PBXIP1, KRT2, CHD4
cytosol	5829	2.23	4.42E-04	AP2A2, CCNB2, PLEC, FLNA, SEC16A, SRP72, HBA1, HBA1, POMP, AHCTF1, APOB, OAS2, ENDOG, ACIN1, DCTD, APP, HERC4, DHX36, DDX58, CASP1, ZFP36L1, PDCD4, ASS1, AHNAK, FLNB, ISYNA1, MAP2K4, ABHD14B, FBP1, PYCARD
**Helium/nitrogen plasma**				
pore complex	46930	13.78	4.18E-02	TPR, AHCTF1, NUP214, RANBP2, C6
chromosomal region	98687	7.83	3.00E-03	MKI67, TPR, WDR43, AHCTF1, RIF1, INCENP, PRKDC, TP53BP1, TNKS1BP1
focal adhesion	5925	5.97	8.72E-03	PLEC, FLNA, PTK7, SDC4, NUP214, AHNAK, FLNB, PLAUR, TNC, MDC1
cell junction	30054	3.24	3.48E-02	LYN, ECT2, PLEC, FLNA, PTK7, ATAD1, SDC4, PKP1, NUP214, AHNAK, FLNB, PLAUR, STX3, SPTAN1, TNC, MDC1
intracellular non-membrane-bounded organelle	43232	2.45	1.79E-05	DDX54, MKI67, ECT2, PLEC, FLNA, ID1, TPR, HBA1, RRP1, HBA1, WDR43, SCAF11, MBD2, AHCTF1, DNTTIP2, WDR46, ACIN1, TCOF1, SDC4, PKP1, POLR1A, TRIM21, EPPK1, AHNAK, DIDO1, CEP170B, FLNB, ABHD14B, UBR4, RIF1
cytosol	5829	2.19	1.84E-02	LYN, ECT2, PLEC, FLNA, SEC16A, IFIT1, HBA1, HBA1, POMP, FTH1, AHCTF1, OAS2, ENDOG, ARFIP1, ACIN1, CASP1, TRIM21, PPM1A, AHNAK, NUP214, FLNB, ISYNA1, MX1, ABHD14B, RANBP2, DYNC1H1, PLEK, SPTAN1, INCENP, PRKDC
nucleus	5634	1.83	2.24E-04	DDX54, CDK7, LYN, MKI67, ECT2, FAM175B, FLNA, ID1, TPR, RRP1, WDR43, POMP, SCAF11, MBD2, FTH1, AHCTF1, TMEM109, OAS2, ENDOG, DNTTIP2, WDR46, ACIN1, TCOF1, SRRM2, APLP2, PKP1, POLR1A, UBE2O, TRIM21, HDAC7
**KEGG pathway**		**GO number**	***p*** **value**	**Molecules**
**Helium plasma**				
Extracellular matrix (ECM)-receptor interaction		4512	6.40E-7	SDC4, CD44, LAMB3, TNC, THBS1, LAMC2, LAMA3
Amoebiasis		5146	3.26E-5	SERPINB13, SERPINB3, LAMB3, C8B, LAMA3, LAMC2
Salmonella infection		5132	1.10E-4	CASP1, PYCARD, FLNB, FLNA, DYNC1H1
Focal adhesion		4510	1.82E-4	FLNB, LAMB3, TNC, FLNA, LAMA3, THBS1, LAMC2
Proteoglycans in cancer		5205	2.89E-4	PDCD4, FLNB, PLAUR, SDC4, CD44, FLNA, THBS1
Complement and coagulation cascades		4610	6.65E-4	F10, PLAUR, C6, C8B
**Helium/nitrogen plasma**				
Salmonella infection		5132	4.69E-4	CASP1, FLNB, FLNA, DYNC1H1
Viral carcinogenesis		5203	1.29E-3	LYN, UBR4, HDAC7, HLA-C, CHD4
Complement and coagulation cascades		4610	3.46E-3	F10, PLAUR, C6
RNA transport		3013	4.64E-3	TPR, ACIN1, RANBP2, NUP214
Extracellular matrix (ECM)-receptor interaction		4512	6.47E-3	SDC4, LAMB3, TNC
Focal adhesion		4510	1.27E-2	FLNB, LAMB3, TNC, FLNA
**Helium/oxygen plasma**				
Mineral absorption		4978	1.76E-3	ATP2B1, FTH1

Top biological processes, cellular components and KEGG pathways altered by the different plasma treatments (helium, He; helium/nitrogen, N; helium/oxygen, O) in comparison to control HaCaT cells (C), analyzed by Panther (http://pantherdb.org) and by STRING (http://string-db.org/).
