# Supplementary material for: Mechanistic insights into the impact of Cold Atmospheric Pressure Plasma on human epithelial cell lines
Source: Sci Rep. 2017 Jan 25;7:41163. doi: 10.1038/srep41163 (PMC5264585; doi:10.1038/srep41163)
Supplement: Supplementary Data [file srep41163-s1.pdf]

Mechanistic insights into the impact of Cold Atmospheric Pressure Plasma on human epithelial cell lines

Marlène Dezest [a], Laurent Chavatte [a], Marion Bourdens[b], Damien Quinton[c], Mylène Camus[d], Luc Garrigues[d], Pascal Descargues [e], Stéphane Arbault[c], Odile Burlet-Schiltz[d], Louis Casteilla [b] Franck Clément[a], Valérie Planat[b&] and Anne-Laure Bulteau [a&*]

Supplementary Methods

Determination of cytosolic glutathione levels (GSH)

Glutathione levels were determined using Thiol tracker violet detection reagent ((Thermofischer, Saint Aubin, France) and analyzed using a microplate fluorimetric reader (BMG-FLUOstar Galaxy, Stuttgart, Germany), excitation/emission wavelengths were 405/526 nm.

Assay for aconitase activity

Mitochondria were isolated as previously described [25]. Mitochondria were suspended in 25 mM phosphate buffer pH 7.25 supplemented with 0.05% Triton X-100 and aconitase activity was assayed spectrophotometrically at 340 nm, as previously described [26].

Assay for KGDH

Mitochondria were diluted in 25 mM MOPS, 0.05% Triton X-100, pH 7.4, and KGDH activity was assayed spectrophotometrically as the rate of NAD^+ reduction to NADH as previously described [27].

Supplementary Figure Legends

Fig. S1. Antioxidant response following plasma treatment.

A. Measurement of reduced glutathione levels. HaCaT, SK-MEL-28 and HCT-116 cells were exposed to plasma treatment (He, He-O_2 and He-N_2) for 5 min with 1 hr post-treatment

storage. 24 hr after plasma treatment they were incubated with ThiolTrackerTM Violet dye for 30 min and GSH level was assayed using a microplate fluorimetric reader. GSH level is presented as a percent of non-treated cells. Data, mean ± SEM from three independent cultures,* $P < 0.05$; B. NRF2 induction following plasma treatment. HaCaT and HCT-116 cells were exposed to plasma treatment (He, He-O_2 and He-N_2) for 5 min with 1 hr post-treatment storage. 24 hr after plasma treatment, NRF2 expression was detected by immunoblotting against NRF2 protein using whole-cell lysates (n = 3). C. Actin was used as loading control for quantification. D. Removal of extracellular H_2O_2 by cells is a first-order process. HaCaT cells were grown in normal or medium supplemented in Selenium (Se), a condition which is known to increase glutathione peroxidase activity, and exposed to plasma treatment (He, He-O_2 and He-N_2) for 5 min and then assayed for the remaining extracellular H_2O_2 by cyclic voltammetry or Amplex red assays. The observed first-order rate constant for the loss of extracellular H_2O_2 in this experiment was determined to be kobs= 0.31 min^{-1} for He-plasma, kobs= 0,28 min^{-1} for He-N_2 plasma, kobs= 1,04 min^{-1} He-O_2 plasma and kobs= 0,72 min^{-1} for HaCaT cell supplemented with selenium and treated with He-plasma. HCT-116 and SK-MEL-28 cells were grown in normal medium and exposed to He plasma treatment and then assayed for the remaining extracellular H_2O_2 by Amplex red assays. The observed first-order rate constant for the loss of extracellular H_2O_2 in this experiment was determined to be kobs= 0.78 min^{-1} for HCT-116 cells and kobs= 1,08 min^{-1} for SK-MEL-28 cells.

Fig. S2. Effect of plasma treatment on activity of key metabolic enzymes in HaCaT cells. A. HaCaT cells were exposed to PAL for 1 hr (PBS treated for 5 min by He, He-O_2 and He-N_2 plasmas). Mitochondria were prepared 24hr after plasma treatment and aconitase activity was assayed. B. For the same CAPP treatment, mitochondria were prepared 24 hr plasma

treatment and α ketoglutarate activity was assayed. Data, mean ± SEM from three independent cultures,* $P < 0.05$; ** $P < 0.01$.

Table S1. List of proteins of varying abundances after the different plasma treatments (helium, He; helium/nitrogen, N; helium/oxygen, O) in comparison with control HaCaT cells (C)

Table S2. List of proteins affected by the plasma activated treatments in HaCaT cells comparison with control cells.

Supplementary Figure 1

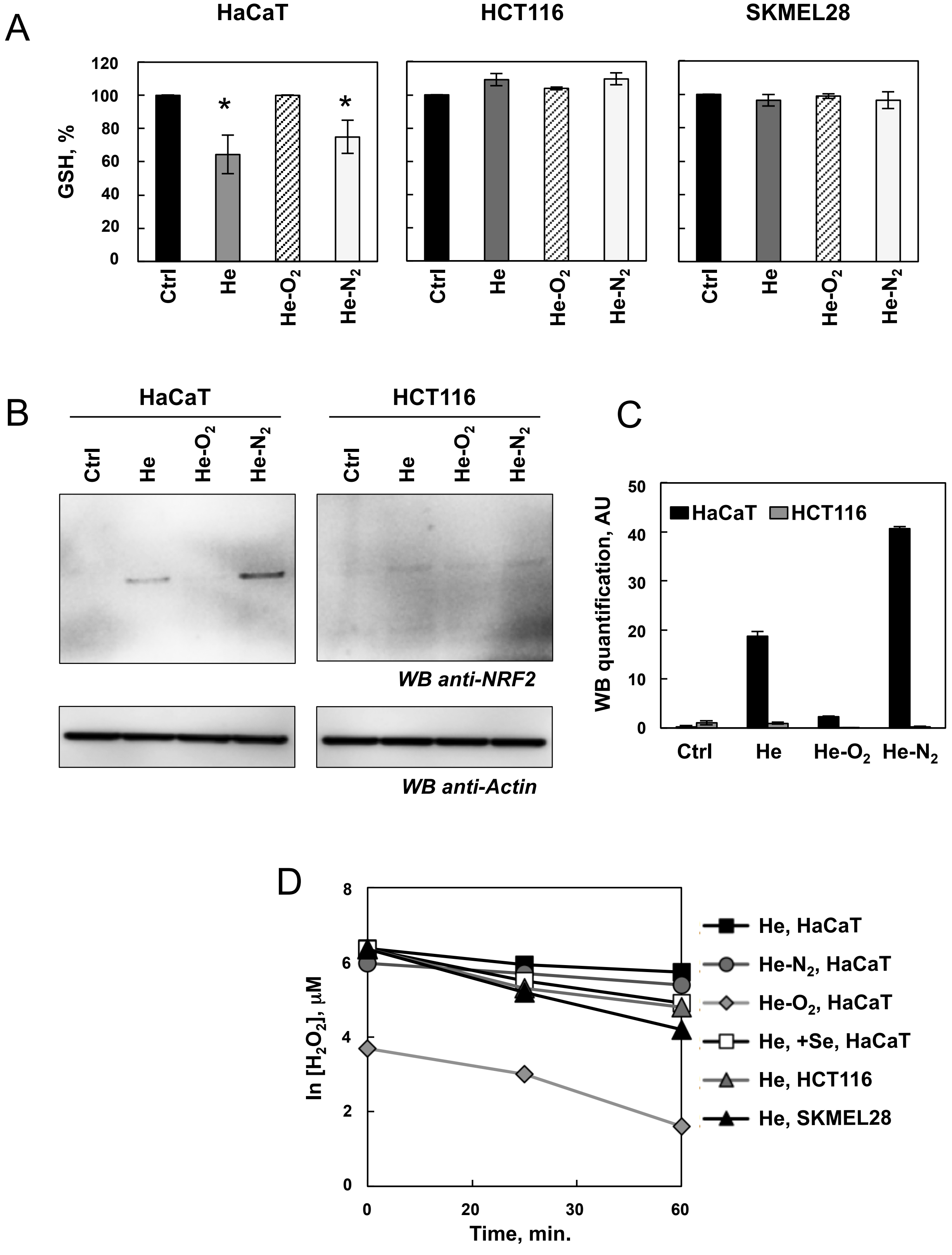

Supplementary Figure 2

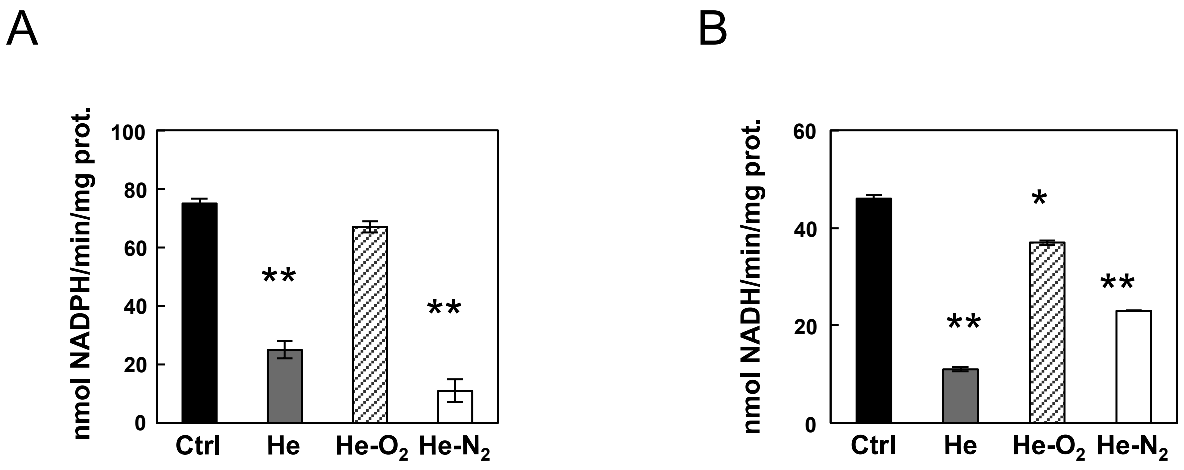

Table S1. List of proteins altered by the different plasma treatments (helium, He; helium/nitrogen, N; helium/oxygen, O) in comparison to control HaCaT cells (C)

Gene names	Acc n°	Name	Ratio N/C	*p* value	Ratio He/C	*p* value	Ratio O2/C	*p* value
**Up and down-regulated in three plasma treatments**								
**AARS2**	Q5JTZ9	Alanine--tRNA ligase, mitochondrial	1334,94	0,0000	1299,55	0,0000	885,55	0,0001
**SUPT6H**	Q7KZ85	Transcription elongation factor SPT6	14,67	0,0000	17,70	0,0002	14,90	0,0002
**KRT1**	P04264	Keratin, type II cytoskeletal 1	11,23	0,0050	4,26	0,0008	3,00	0,0023
**PLAUR**	Q03405	Urokinase plasminogen activator surface receptor	9,75	0,0000	12,19	0,0004	5,34	0,0011
**DDX54**	Q8TDD1	ATP-dependent RNA helicase DDX54	3,45	0,0039	4,39	0,0017	0,20	0,0013
**ASPH**	Q12797	Aspartyl/asparaginyl beta-hydroxylase	2,21	0,0016	2,54	0,0065	2,12	0,0062
**OAS2**	P29728	2-5-oligoadenylate synthase 2	0,28	0,0006	0,37	0,0041	0,47	0,0094
**Up and down-regulated in two plasma treatments**								
**CHD4**	Q14839	Chromodomain-helicase-DNA-binding protein 4	111,08	0,0001	118,18	0,0001		
**POMP**	Q9Y244	Proteasome maturation protein	29,85	0,0002	29,78	0,0002		
**TP53BP1**	Q12888	Tumor suppressor p53-binding protein 1	27,27	0,0000	23,30	0,0000		
**MKI67**	P46013	Antigen KI-67	21,77	0,0007	22,59	0,0009		
**FLNB**	O75369	Filamin-B	19,15	0,0000	19,61	0,0000		
**TPR**	P12270	Nucleoprotein TPR	15,95	0,0001	18,24	0,0001		
**FLNA**	P21333	Filamin-A	14,60	0,0001	15,73	0,0001		
**DYNC1H1**	Q14204	Cytoplasmic dynein 1 heavy chain 1	14,16	0,0003	14,98	0,0003		
**F10**	P00742	Coagulation factor X	12,73	0,0001	21,30	0,0000		
**PRKDC**	P78527	DNA-dependent protein kinase catalytic subunit	12,72	0,0002	12,22	0,0003		
**C6**	P13671	Complement component C6	12,21	0,0002	19,78	0,0001		
**HLA-C**	P04222	HLA class I histocompatibility antigen, Cw-3 alpha chain	11,27	0,0000			12,08	0,0000
**DIDO1**	Q9BTC0	Death-inducer obliterator 1	9,50	0,0000	9,04	0,0001		
**PRRC2C**	Q9Y520	Protein PRRC2C	9,29	0,0002	11,06	0,0008		
**RANBP2**	P49792	E3 SUMO-protein ligase RanBP2	9,03	0,0004	10,75	0,0005		
**LGMN**	Q99538	Legumain	8,41	0,0043	9,19	0,0047		
**MDC1**	Q14676	Mediator of DNA damage checkpoint protein 1	7,57	0,0025	9,12	0,0028		
**WDR46**	O15213	WD repeat-containing protein 46	7,56	0,0009	5,81	0,0005		
**PLEC**	Q15149	Plectin	7,42	0,0001	8,07	0,0001		
**SPTAN1**	Q13813	Spectrin alpha chain, non-erythrocytic 1	6,24	0,0033	5,87	0,0040		
**SDC4**	P31431	Syndecan-4	6,18	0,0013	7,11	0,0015		
**AHCTF1**	Q8WYP5	Protein ELYS	6,09	0,0001	5,14	0,0004		
**EPPK1**	P58107	Epiplakin	5,81	0,0012	8,15	0,0008		
**UBE2O**	Q9C0C9	Ubiquitin-conjugating enzyme E2 O	5,14	0,0054	4,10	0,0084		
**SEC16A**	O15027	Protein transport protein Sec16A	5,09	0,0020	4,21	0,0078		
**TNKS1BP1**	Q9C0C2	182 kDa tankyrase-1-binding protein	4,70	0,0005	4,39	0,0007		
**SRRM2**	Q9UQ35	Serine/arginine repetitive matrix protein 2	4,60	0,0000	8,27	0,0002		
**TNC**	P24821	Tenascin	4,20	0,0020	8,35	0,0086		
**FAM175B**	Q15018	BRISC complex subunit Abro1	4,05	0,0058	3,06	0,0001		
**MBD2**	Q9UBB5	Methyl-CpG-binding domain protein 2	3,98	0,0030	4,36	0,0020		
**HBA1**	P69905	Hemoglobin subunit alpha	3,95	0,0021	12,17	0,0000		
**CLDN4/9/6/3**	O14493	Claudin-4/9/6/3	3,72	0,0003			2,92	0,0007
**GOLM1**	Q8NBJ4	Golgi membrane protein 1	3,65	0,0001	4,75	0,0015		
**ECT2**	Q9H8V3	Protein ECT2	3,60	0,0033			2,93	0,0074
**CEP170B**	Q9Y4F5	Centrosomal protein of 170 kDa protein B	3,46	0,0097	7,16	0,0067		
**AHNAK**	Q09666	Neuroblast differentiation-associated protein AHNAK	3,43	0,0002	4,25	0,0001		
**SLC30A1**	Q9Y6M5	Zinc transporter 1	3,21	0,0001	5,30	0,0001		
**CHD8/6/9/7**	Q9HCK8	Chromodomain-helicase-DNA-binding protein 8/6/9/7	3,03	0,0002	2,65	0,0009		
**TCOF1**	Q13428	Treacle protein	3,02	0,0051	3,32	0,0021		
**MARCKSL1**	P49006	MARCKS-related protein	2,77	0,0024	2,72	0,0019		
**UBR4**	Q5T4S7	E3 ubiquitin-protein ligase UBR4	2,52	0,0004	2,97	0,0029		
**HABP2**	Q14520	Hyaluronan-binding protein 2	2,51	0,0002	3,84	0,0003		
**RAP2B/A**	P61225	Ras-related protein Rap-2b/a	2,46	0,0051			2,05	0,0095
**ACIN1**	Q9UKV3	Apoptotic chromatin condensation inducer in the nucleus	2,44	0,0054	2,40	0,0076		
**WDR43**	Q15061	WD repeat-containing protein 43	2,29	0,0010	2,10	0,0018		
**LAMB3**	Q13751	Laminin subunit beta-3	2,18	0,0002	3,59	0,0001		
**ATAD1**	Q8NBU5	ATPase family AAA domain-containing protein 1	2,07	0,0031	3,31	0,0081		
**FTH1**	P02794	Ferritin heavy chain	2,04	0,0038			2,28	0,0028
**IVL**	P07476	Involucrin	0,50	0,0029	0,48	0,0088		
**CD109**	Q6YHK3	CD109 antigen	0,47	0,0013	0,43	0,0081		
**ZZEF1**	O43149	Zinc finger ZZ-type and EF-hand domain-containing protein 1	0,44	0,0005	0,42	0,0053		
**PKP1**	Q13835	Plakophilin-1	0,38	0,0004	0,37	0,0005		
**ISYNA1**	Q9NPH2	Inositol-3-phosphate synthase 1	0,26	0,0035	0,19	0,0069		
**PTK7**	Q13308	Inactive tyrosine-protein kinase 7	0,25	0,0029	0,20	0,0084		
**ABHD14B**	Q96IU4	Alpha/beta hydrolase domain-containing protein 14B	0,24	0,0061	0,24	0,0056		
**REPS1**	Q96D71	RalBP1-associated Eps domain-containing protein 1	0,24	0,0000	0,23	0,0000		
**ENDOG**	Q14249	Endonuclease G, mitochondrial	0,10	0,0000	0,08	0,0037		
**CASP1**	P29466	Caspase-1;Caspase-1 subunit p20;Caspase-1 subunit p10	0,09	0,0000	0,14	0,0045		
**KRT2**	P35908	Keratin, type II cytoskeletal 2 epidermal			3,79	0,0002	2,71	0,0046
**Up and down-regulated in only one plasma treatment**								
**HDAC7**	Q8WUI4	Histone deacetylase 7	61,34	0,0084				
**NUP214**	P35658	Nuclear pore complex protein Nup214	7,26	0,0041				
**DNTTIP2**	Q5QJE6	Deoxynucleotidyltransferase terminal-interacting protein 2	5,05	0,0062				
**INCENP**	Q9NQS7	Inner centromere protein	4,91	0,0074				
**HECTD1**	Q9ULT8	E3 ubiquitin-protein ligase HECTD1	4,84	0,0053				
**PLEK**	P08567	Pleckstrin	4,70	0,0061				
**RIF1**	Q5UIP0	Telomere-associated protein RIF1	4,69	0,0018				
**SCAF11**	Q99590	Protein SCAF11	4,68	0,0065				
**LYN**	P07948	Tyrosine-protein kinase Lyn	4,57	0,0006				
**PRPF4B**	Q13523	Serine/threonine-protein kinase PRP4 homolog	3,14	0,0002				
**PPM1A**	P35813	Protein phosphatase 1A	3,13	0,0060				
**CDK7**	P50613	Cyclin-dependent kinase 7	3,06	0,0001				
**DLGAP4**	Q9Y2H0	Disks large-associated protein 4	2,85	0,0002				
**TMEM109**	Q9BVC6	Transmembrane protein 109	2,71	0,0013				
**APOBEC3B**	Q9UH17	DNA dC->dU-editing enzyme APOBEC-3B	2,69	0,0005				
**STX3**	Q13277	Syntaxin-3	2,58	0,0004				
**POLR1A**	O95602	DNA-directed RNA polymerase I subunit RPA1	2,43	0,0054				
**APLP2**	Q06481	Amyloid-like protein 2	2,15	0,0057				

**TNPO2**	O14787	Transportin-2	2,08	0,0038				
**GDAP1**	Q8TB36	Ganglioside-induced differentiation-associated protein 1	2,01	0,0058				
**RRP1**	P56182	Ribosomal RNA processing protein 1 homolog A	2,00	0,0081				
**IFIT1**	P09914	Interferon-induced protein with tetratricopeptide repeats 1	0,48	0,0007				
**EFNB1**	P98172	Ephrin-B1	0,37	0,0001				
**MX1**	P20591	Interferon-induced GTP-binding protein Mx1	0,36	0,0002				
**IDH2**	P48735	Isocitrate dehydrogenase [NADP], mitochondrial	0,35	0,0093				
**ID1**	P41134	DNA-binding protein inhibitor ID-1	0,34	0,0098				
**ARFIP1**	P53367	Arfaptin-1	0,28	0,0022				
**TRIM21**	P19474	E3 ubiquitin-protein ligase TRIM21	0,27	0,0086				
**DHRS1**	Q96LJ7	Dehydrogenase/reductase SDR family member 1	0,12	0,0009				
**C8B**	P07358	Complement component C8 beta chain			12,47	0,0003		
**IGF2R**	P11717	Cation-independent mannose-6-phosphate receptor			9,25	0,0003		
**QIL1**	Q5XKP0	Protein QIL1			6,49	0,0030		
**LHFPL2**	Q6ZUX7	Lipoma HMGIC fusion partner-like 2 protein			5,06	0,0010		
**DCTD**	P32321	Deoxycytidylate deaminase			4,92	0,0003		
**VTN**	P04004	Vitronectin			4,78	0,0003		
**PRPF8**	Q6P2Q9	Pre-mRNA-processing-splicing factor 8			4,44	0,0097		
**APOM**	O95445	Apolipoprotein M			3,70	0,0001		
**EHD3**	Q9NZN3	EH domain-containing protein 3			3,65	0,0049		
**POLR1B**	Q9H9Y6	DNA-directed RNA polymerase I subunit RPA2			3,61	0,0092		
**CEP164**	Q9UPV0	Centrosomal protein of 164 kDa			3,60	0,0000		
**ZC3H4**	Q9UPT8	Zinc finger CCCH domain-containing protein 4			3,47	0,0025		
**RRAGA/B**	Q7L523	Ras-related GTP-binding protein A/B			3,20	0,0056		
**RNF19B/A**	Q6ZMZ0	E3 ubiquitin-protein ligase RNF19B/A			3,13	0,0018		
**APOB**	P04114	Apolipoprotein B-100;Apolipoprotein B-48			2,78	0,0065		
**SYNE2**	Q8WXH0	Nesprin-2			2,67	0,0022		
**APP**	P05067	Amyloid beta A4 protein			2,67	0,0092		
**LAMA3**	Q16787	Laminin subunit alpha-3			2,66	0,0000		
**LAMC2**	Q13753	Laminin subunit gamma-2			2,65	0,0007		
**FTSJ3**	Q8IY81	pre-rRNA processing protein FTSJ3			2,47	0,0011		
**THBS1**	P07996	Thrombospondin-1			2,30	0,0008		
**QRICH1**	Q2TAL8	Glutamine-rich protein 1			2,26	0,0058		
**CD44**	P16070	CD44 antigen			2,26	0,0081		
**TIMM21**	Q9BVV7	Mitochondrial import inner membrane translocase subunit Tim21			2,21	0,0059		
**MTHFD2**	P13995	Bifunctional methylenetetrahydrofolate dehydrogenase/cyclohydrolase			2,09	0,0073		
**ZFP36L1**	Q07352	Zinc finger protein 36			2,08	0,0055		
**SRP72**	O76094	Signal recognition particle subunit SRP72			0,50	0,0008		
**PBXIP1**	Q96AQ6	Pre-B-cell leukemia transcription factor-interacting protein 1			0,49	0,0013		
**HMGN1**	P05114	Non-histone chromosomal protein HMG-14			0,49	0,0002		
**OAS3**	Q9Y6K5	2-5-oligoadenylate synthase 3			0,48	0,0052		
**SERPINB13**	Q9UIV8	Serpin B13			0,48	0,0006		
**STK39**	Q9UEW8	STE20/SPS1-related proline-alanine-rich protein kinase			0,47	0,0021		
**FBP1**	P09467	Fructose-1,6-bisphosphatase 1			0,47	0,0006		
**DHX36**	Q9H2U1	Probable ATP-dependent RNA helicase DHX36			0,47	0,0029		
**LYPLA2**	O95372	Acyl-protein thioesterase 2			0,46	0,0047		
**PYCARD**	Q9ULZ3	Apoptosis-associated speck-like protein containing a CARD			0,46	0,0004		
**LCLAT1**	Q6UWP7	Lysocardiolipin acyltransferase 1			0,45	0,0004		
**RABL6**	Q3YEC7	Rab-like protein 6			0,45	0,0036		
**ETHE1**	O95571	Persulfide dioxygenase ETHE1, mitochondrial			0,44	0,0029		
**NAPRT1**	Q6XQN6	Nicotinate phosphoribosyltransferase			0,44	0,0083		
**ASS1**	P00966	Argininosuccinate synthase			0,43	0,0003		
**AP2A2**	O94973	AP-2 complex subunit alpha-2			0,41	0,0022		
**SRP19**	P09132	Signal recognition particle 19 kDa protein			0,41	0,0021		
**NT5DC1**	Q5TFE4	5-nucleotidase domain-containing protein 1			0,41	0,0052		
**HLA-DRA**	P01903	HLA class II histocompatibility antigen, DR alpha chain			0,40	0,0051		
**NT5C2**	P49902	Cytosolic purine 5-nucleotidase			0,40	0,0001		
**LRRC1**	Q9BTT6	Leucine-rich repeat-containing protein 1			0,40	0,0049		
**LRSAM1**	Q6UWE0	E3 ubiquitin-protein ligase LRSAM1			0,38	0,0058		
**ASUN**	Q9NVM9	Protein asunder homolog			0,31	0,0023		
**SERPINB3**	P29508	Serpin B3			0,29	0,0000		
**POLR2G**	P62487	DNA-directed RNA polymerase II subunit RPB7			0,29	0,0051		
**HERC4**	Q5GLZ8	Probable E3 ubiquitin-protein ligase HERC4			0,27	0,0049		
**MAP2K4**	P45985	Dual specificity mitogen-activated protein kinase kinase 4			0,26	0,0032		
**FANCD2**	Q9BXW9	Fanconi anemia group D2 protein			0,26	0,0066		
**CCNB2**	O95067	G2/mitotic-specific cyclin-B2			0,25	0,0100		
**DDX58**	O95786	Probable ATP-dependent RNA helicase DDX58			0,21	0,0067		
**UFC1**	Q9Y3C8	Ubiquitin-fold modifier-conjugating enzyme 1			0,21	0,0007		
**PDCD4**	Q53EL6	Programmed cell death protein 4			0,19	0,0054		
**ABCB6**	Q9NP58	ATP-binding cassette sub-family B member 6, mitochondrial			0,19	0,0004		
**AAR2**	Q9Y312	Protein AAR2 homolog			0,10	0,0062		
**NUCKS1**	Q9H1E3	Nuclear ubiquitous casein and cyclin-dependent kinase substrate 1			0,07	0,0000		
**GAK**	O14976	Cyclin-G-associated kinase					9,54	0,0004
**RANBP3**	Q9H6Z4	Ran-binding protein 3					6,18	0,0086
**ZRANB2**	O95218	Zinc finger Ran-binding domain-containing protein 2					4,23	0,0039
**DPF2**	Q92785	Zinc finger protein ubi-d4					3,72	0,0007
**BRD4**	O60885	Bromodomain-containing protein 4					3,16	0,0018
**THADA**	Q6YHU6	Thyroid adenoma-associated protein					2,47	0,0094
**HTRA2**	O43464	Serine protease HTRA2, mitochondrial					2,36	0,0018
**DCTN4**	Q9UJW0	Dynactin subunit 4					2,31	0,0013
**ATP2B1/3**	P20020	Plasma membrane calcium-transporting ATPase 1/3					2,17	0,0037
**CDYL**	Q9Y232	Chromodomain Y-like protein					2,08	0,0008
**MYBBP1A**	Q9BQG0	Myb-binding protein 1A					0,46	0,0040
**ODR4**	Q5SWX8	Protein odr-4 homolog					0,34	0,0098
**MPHOSPH8**	Q99549	M-phase phosphoprotein 8					0,32	0,0026
**C1orf174**	Q8IYL3	UPF0688 protein C1orf174					0,29	0,0006
**PPIC**	P45877	Peptidyl-prolyl cis-trans isomerase C					0,20	0,0000
**HLA-C**	Q07000	HLA class I histocompatibility antigen, Cw-15 alpha chain					0,16	0,0000

Table S2. List of proteins affected by the plasma activated treatments in HaCaT cells comparison to control cells

Gene names	Protein names	Acc n°	Ratio He/C	t-test
**Up-regulated proteins**				
**Q16763**	Ubiquitin-conjugating enzyme E2 S	Q5JTZ9	34,37	0,0000
**Q4G0Z9**	MCM domain-containing protein 2	Q14839	23,31	0,0007
**P42357**	Histidine ammonia-lyase	Q9Y244	20,19	0,0000
**Q96CM8**	Acyl-CoA synthetase family member 2, mitochondrial	Q12888	9,88	0,0000
**Q8IZP0**	Abl interactor 1	P46013	8,46	0,0045
**Q96EM0**	Trans-L-3-hydroxyproline dehydratase	P00742	7,24	0,0056
**O14656**	Torsin-1A	P13671	2,56	0,0025
**Q16787**	Laminin subunit alpha-3	O75369	2,17	0,0089
**Q96JB1**	Dynein heavy chain 8, axonemal	P12270	2,16	0,0053
**P30307**	M-phase inducer phosphatase 3	Q7KZ85	2,16	0,0083
**Down-regulated proteins**				
**P52292**	Importin subunit alpha-1	KPNA2	0,48	0,0016
**P20340**	Ras-related protein Rab-6A	RAB6A	0,47	0,0000
**Q5K651**	Sterile alpha motif domain-containing protein 9	SAMD9	0,43	0,0029
**O75449**	Katanin p60 ATPase-containing subunit A1	KATNA1	0,43	0,0014
**P50281**	Matrix metalloproteinase-14	MMP14	0,43	0,0044
**P49768**	Presenilin-1	PSEN1	0,41	0,0062
**Q9BPZ7**	Target of rapamycin complex 2 subunit MAPKAP1	MAPKAP1	0,37	0,0020
**Q9NQW6**	Actin-binding protein anillin	ANLN	0,37	0,0053
**Q7Z412**	Peroxisome assembly protein 26	PEX26	0,34	0,0095
**Q6UXN9**	WD repeat-containing protein 82	WDR82	0,33	0,0020
**Q13835**	Plakophilin-1	PKP1	0,32	0,0057
**Q9NUQ3**	Gamma-taxilin	TXLNG	0,31	0,0010
**P43003**	Excitatory amino acid transporter 1	SLC1A3	0,25	0,0067
**O95155**	Ubiquitin conjugation factor E4 B	UBE4B	0,23	0,0000
**Q9H4H8**	Protein FAM83D	FAM83D	0,22	0,0072
**Q53EL6**	Programmed cell death protein 4	PDCD4	0,21	0,0010
**Q6NXE6**	Armadillo repeat-containing protein 6	ARMC6	0,20	0,0036
**Q96TA2**	ATP-dependent zinc metalloprotease YME1L1	YME1L1	0,18	0,0075
**Q4J6C6**	Prolyl endopeptidase-like	PREPL	0,14	0,0019
**P40937**	Replication factor C subunit 5	RFC5	0,13	0,0007
**Q07000**	HLA class I histocompatibility antigen	HLA-C	0,11	0,0033
**I1YAP6**	Tripartite motif-containing protein 77	TRIM77	0,11	0,0004
**Q96KB5**	Lymphokine-activated killer T-cell-originated protein kinase	PBK	0,09	0,0000
**P42695**	Condensin-2 complex subunit D3	NCAPD3	0,02	0,0036
**Q9BUP3**	Oxidoreductase HTATIP2	HTATIP2	0,01	0,0002
